# Maximum Entropy for the International Division of Labor

**DOI:** 10.1371/journal.pone.0129955

**Published:** 2015-07-14

**Authors:** Hongmei Lei, Ying Chen, Ruiqi Li, Deli He, Jiang Zhang

**Affiliations:** School of Systems Science, Beijing Normal University, Beijing, China; East China University of Science and Technology, CHINA

## Abstract

As a result of the international division of labor, the trade value distribution on different products substantiated by international trade flows can be regarded as one country’s strategy for competition. According to the empirical data of trade flows, countries may spend a large fraction of export values on ubiquitous and competitive products. Meanwhile, countries may also diversify their exports share on different types of products to reduce the risk. In this paper, we report that the export share distribution curves can be derived by maximizing the entropy of shares on different products under the product’s complexity constraint once the international market structure (the country-product bipartite network) is given. Therefore, a maximum entropy model provides a good fit to empirical data. The empirical data is consistent with maximum entropy subject to a constraint on the expected value of the product complexity for each country. One country’s strategy is mainly determined by the types of products this country can export. In addition, our model is able to fit the empirical export share distribution curves of nearly every country very well by tuning only one parameter.

## Introduction

Dating back to Adam Smith, the division of labor had been an important topic in economic literature [1–5] because the level of the division of labor determines a nation’s efficiency of wealth accumulation. The importance of the division of labor in today’s capitalist free market system is beyond question: people must do what they are good at to survive today’s competition [1]. In particular, as the process of globalization accelerates, the division of labor occurs over a much larger scale – the international trade market [6–9]. In this market, all countries face similar challenges [10–13]: they must invest their resources and technologies on the development of competitive products because they know “a Jack of all trades is a master of none”. On the contrary, each country must diversify its exports on a wide variety of products to avoid “putting all his eggs in one basket” [14]. Nations currently face such a dilemma: if too specialized, they may not be robust enough and suffer from financial crises; if too diversified, they may fail to gain a competitive advantage. Thus, there is a trade-off between these two extreme strategies.

As a result of this trade-off, the ranked export share distribution curve for each country, which is available from highly detailed trade flow data, can be regarded as the substantiation of the international division of labor. For example, the United States has an inverse “S” curve with a high peak at the electronic microcircuits and vehicles parts, whereas Gabon has a much flatter “L” curve, with a relatively high peak at crude petroleum and logging (see Figure A and B in S1 File). Our hypothesis is that both the economic profit and the real conditions of the country in the global market determine the shape of the export share curve. It is very important for one country to export the appropriate types of products and in the correct proportion. In addition, the realized ranked share distribution curves should be the result of a long term optimization.

By what principle is the export strategy optimized? A promising answer provided by this paper is maximizing information entropy [14]. The concept of entropy was initially developed in statistical physics and was introduced to communication and information theory by Shannon [15]. Currently, the maximum entropy principle (MEP) has become a powerful interdisciplinary framework that has been widely applied to economics, ecology, cybernetics [14, 16–18].

We use the information entropy of export share distribution to measure the diversity of one country’s export. We assume that each country seeks to diversify their investment on export products under the constraints of technology, natural resources and policies [8]. All of these constraints are quantified by a single measure: the total complexity budget. This measure is related to the types of products each country can produce. Some high-tech products, such as Google Glass and satellites, require complex production processes, and only a few countries that have a high level of complexity budget can produce them; as a result, high-tech products represent a small fraction of gross export [9]. In contrast, products such as apples or rice require a relatively low complexity level so that many countries are capable of investing resources on them. In [9], the complexity level required by one product is proportional to the negative logarithm of its ubiquity (i.e., the number of countries who export this product). In addition, the total complexity budget of one country is determined by the types of products it exports. Therefore, if the bipartite network between countries and products is given, the export share distribution curve of each country can be solved by the standard MEP. Surprisingly, by tuning only one free parameter, our model can fit the empirical results for nearly all countries, especially large nations.

## Results

### Empirical Results

**Fig 1:**
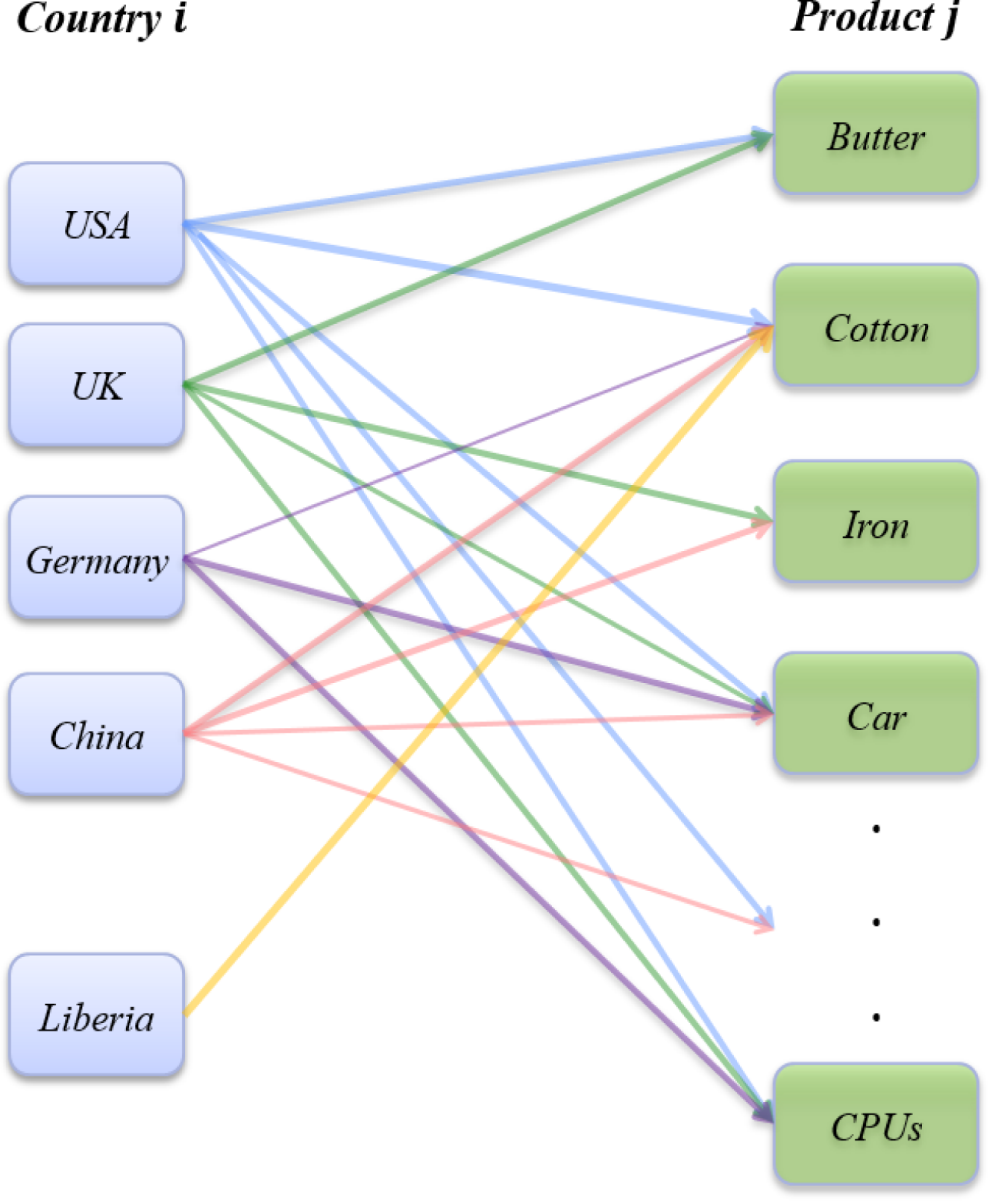
Country-product bipartite network. Country *i*’s diversification *D*_*i*_ is defined as the degree of country *i*. The product *j*’s ubiquity *U*_*i*_ is the degree of *j*. The weight *w*_*ij*_ of the link from country *i* to product *j* denoted the trade flow between them. The ratio *p*_*ij*_ between *w*_*ij*_ and *F*_*i*_, the total trade values of country *i*, is defined as the strategy of *i*.

First, we retrieved the original trade data of all countries from the NBER-UN world trade dataset [19], which includes the detailed bilateral trade flows of approximately 188 countries and 772 types of products (according to the SITC4 classification standard) during 1984–2000. In this paper, we present the representative results of 2000. The best way to illustrate the relationship between countries and products is the country-product bipartite network (see Fig 1), where country *i* links to product *j* if *i* exports *j*. The weight *w*_*ij*_ on the edge ⟨*i*, *j*⟩ represents the volume of trade flow between them. Furthermore, we define the total trade volume (i.e., trade values) of a country as *F*_*i*_.

**Fig 2:**
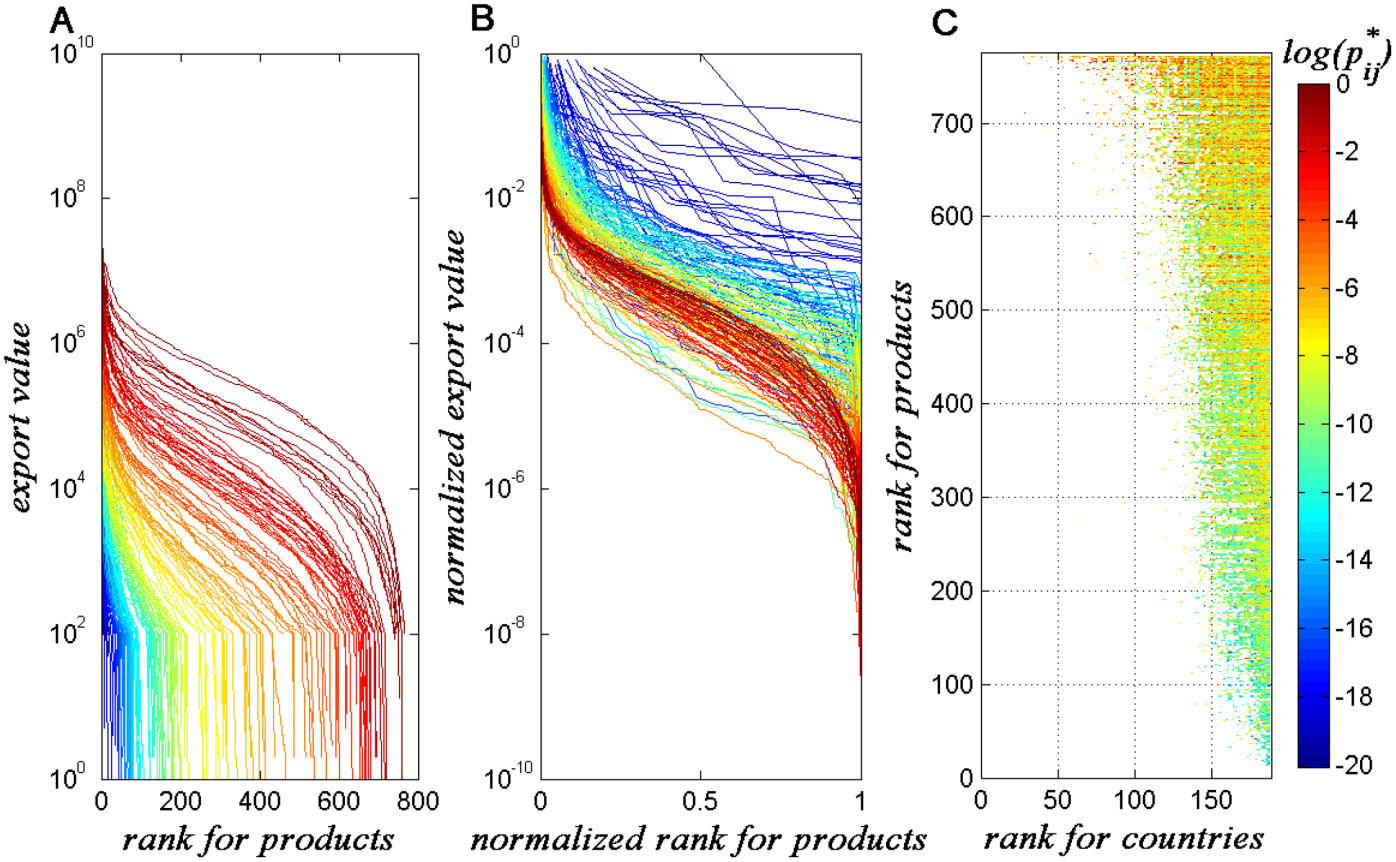
(A) Rank curves of the products trade values for all of the countries coloured by their ascending ranks. The rank curve for products is descending order of its export value in a specific country. (B) Normalized ranked export share curves for all countries coloured by the country’s total export values. (C) The matrix plot of the international division of labor from the empirical data. The horizontal axis represents the ranks of countries sorted by their total export values *F*_*i*_, and the vertical axis represents the ranks of products sorted by their ubiquity worldwide, which is at different scale from (A). The color in each entry represents the logarithmic trade shares $p_{ij}^{*}$ from *i* to *j*. This figure reflects the overall structure of the international division of labor.

Second, we plot the ranked trade value curves for all countries and color them according to their sizes in Fig 2A. We qualitatively observe that all of the curves are of inverted S-shaped and that the curves of large countries are flatter than those of small countries because the types of products (*D*_*i*_) exported by countries increase with the total trade values (*F*_*i*_). This positive correlation between *D*_*i*_ and *F*_*i*_ was previously reported [20] and attributed to the substitutability of different products [21]. An abrupt decrease in the tail part of the curves is observed because many of the trade flows with values less than 100 dollars are missing in the dataset.

To eliminate the size effect and to depict each country’s strategy more clearly, Fig 2B shows the normalized version of Fig 2A. For country *i*, we assign the vertical axis as the empirical share of the product *j* on the total trade value:

$$\begin{array}{c} p_{ij}^{*}=\frac{w_{ij}}{F_{i}}, \end{array}$$ (1)

where the empirical variable $p_{ij}^{*}$ is distinguished by * from the theoretical variables (*p*_*ij*_), which will be introduced in the following sections, and the horizontal axis as *r*_*j*_/*D*_*i*_, where *r*_*j*_ is the ubiquity rank of the product *j* (see Fig 2B). We refer to these curves as the export share distributions $p_{ij}^{*}$, which represent the strategies of the countries. A systematic trend can be qualitatively observed: when the sizes of countries increase, the curves become steeper and transform from L shaped curves to inverse S shaped curves. This observation indicates that small countries always have diversified investments to complement their low diversification level of products, as measured by *D*_*i*_; mid-size countries diversify their exports over a large spectrum of products, while focusing on some products; large countries have a broader spectrum and the export value of minor products changes not that drastically as for small countries.

Finally, to understand the overall situation of international division of labor, we investigate how the normalized weights $p_{ij}^{*}$ are distributed on all country-product pairs by plotting the whole matrix of P* in Fig 2C, in which the rows (products) are sorted by product *j*’s ubiquity *U*_*j*_ (the number of countries exporting product *j* [9, 22]) in an increasing order and the columns (countries) are sorted by country *i*’s diversification *D*_*i*_, the color of each entry represents the logarithm of $p_{ij}^{*}$ from *i* to *j*, and the white areas represent absent country-product pairs. A “triangular structure” [22] can be observed in Fig 2C, which indicates that small countries can export a few products with relatively large ubiquity, whereas large countries have much wider spectra of products. However, when the trading shares are considered (the colors in Fig 2C), we find that high throughput trades (red region) are almost located on the upper-right corner, which indicates large countries actually allocate most of their exports on popular (large ubiquities) products. This observation implies a propensity of risk inversion for large countries—they invest a large amount of exports on products with low complexity and only allocate a small fraction of export on the products with high complexity (see Fig 2A for large countries, the export shares of high complexity product with higher ranks are small).

### Maximum Entropy Model

To understand the underlying mechanism of international division of labor, we present a theoretical model. With given country-product bipartite network, we attempt to derive the export share distribution curves, i.e., *p*_*ij*_ for all *i*, which, roughly speaking, is the strategy of each country. Although the abilities of each country are different. Larger countries generally can produce more complicated products which may be not producible for small countries. For simplicity and generality, we assume all the countries follow some general strategy. In our model, the general strategy is maximizing their entropy under complexity constraints. We apply the maximum entropy framework [16] to derive *p*_*ij*_. According to Shannon [15], the diversity of a country *i*’s export strategy is measured by the Shannon information entropy *H*_*i*_, and

$$\begin{array}{c} H_{i}=-\sum_{j=1}^{D_{i}}p_{ij}\;\ln\;p_{ij}. \end{array}$$ (2)

Next, we present the constraint condition for country *i*, which is a very important and subtle ingredient in MEP [16]. The complexity levels for products are heterogeneous, which means producing and exporting different products may have different costs for country *i*. For example, some complex products, such as Google Glass and rockets, require many capabilities, e.g., rare raw materials, advanced management experiences, high-tech skills [9]. Although only a few countries can produce them, such countries always use only a few export values on these complex products for some consideration. In contrast, some products merely require simple capabilities, and almost all countries can produce and export them. In this paper, we define a product *j*’s complexity as

$$\begin{array}{c} K_{j}=\ln\frac{1}{U_{j}}, \end{array}$$ (3)

where *U*_*j*_ is the ubiquity of product *j*, i.e., how many countries export it. Therefore, the higher the number of countries that export product *j*, the lower is its complexity. By taking a logarithm of 1/*U*_*j*_, our complexity measurement resembles the information unit (bit) (see the second section in S1 File for more discussion). Thus, producing different products may require different complexity levels. Additionally, the total complexity consumption is assumed in our model to balance with the total complexity budget *B*_*i*_ for any country. From empirical study, we find that the complexity budget *B*_*i*_ is proportional to the gross level of complexity for all products that country *i* can export, that is

$$\begin{array}{c} B_{i}=k\sum_{j=1}^{D_{i}}K_{j}. \end{array}$$ (4)

Thus, the complexity budget of *i* is determined by what kind of products *i* can export, i.e., the bipartite network structure. *k* is a free parameter and it is identical for all countries. Finally, country *i* can maximize its information entropy *H*_*i*_ under the constraints of normalization condition of *p*_*ij*_ and the complexity consumption budgets. Thus,

$$\begin{array}{cc} \max & H_{i}=-\sum_{j=1}^{D_{i}}p_{ij}\;\ln\;p_{ij} \end{array}$$ (5)

$$\begin{array}{cc} s.t. & \left\{ \begin{array}{c} \sum_{j=1}^{D_{i}}p_{ij}=1\\ D_{i}\sum_{j=1}^{D_{i}}p_{ij}\;\ln\;\frac{1}{U_{j}}=k\;\sum_{j=1}^{D_{i}}\;\ln\;\frac{1}{U_{j}} \end{array}\right. \end{array}$$ (6)

where the left hand term of the second constraint is $D_{i}\sum_{j=1}^{D_{i}}p_{ij}\;\text{ln}\;\frac{1}{U_{j}}$, which denotes country *i*’s total complexity consumptions, and this summation is weighted by the export share, *p*_*ij*_. While the right hand term of this constraint is country *i*’s total complexity budget, it is determined by *U*_*j*_s, i.e., what kind of products that *i* can export. The constant *k*, the only free parameter in our model, measures the degree to which the complexity budget is influenced by the kinds of products *i* can export. *k* is identical for all countries, therefore, it also measures the sensitivity of export shares with respect to the bipartite network structure in the whole world.

In Eqs (5) and (6), *D*_*i*_ and *U*_*j*_ for all countries and products can be obtained from the empirical bipartite network. Thus, the MEP problem can be solved by the standard Lagrangian multiplier method which will be present in Method section, and the predicted distribution *p*_*ij*_ can be derived. We will compare *p*_*ij*_ with $p_{ij}^{*}$ for all countries. The problem proposed in Eqs (5), (6) is able to recover to a quite classical one with a text solution as first derived by Cover and Thomas [14], if we substitute Cover’s *r*_*i*_(*x*) for *log*(1/*U*_*i*, *j*_) and convert also from Cover’s continuous distribution to discrete distribution.

### Comparison Between the Model and the Empirical Data

**Fig 3:**
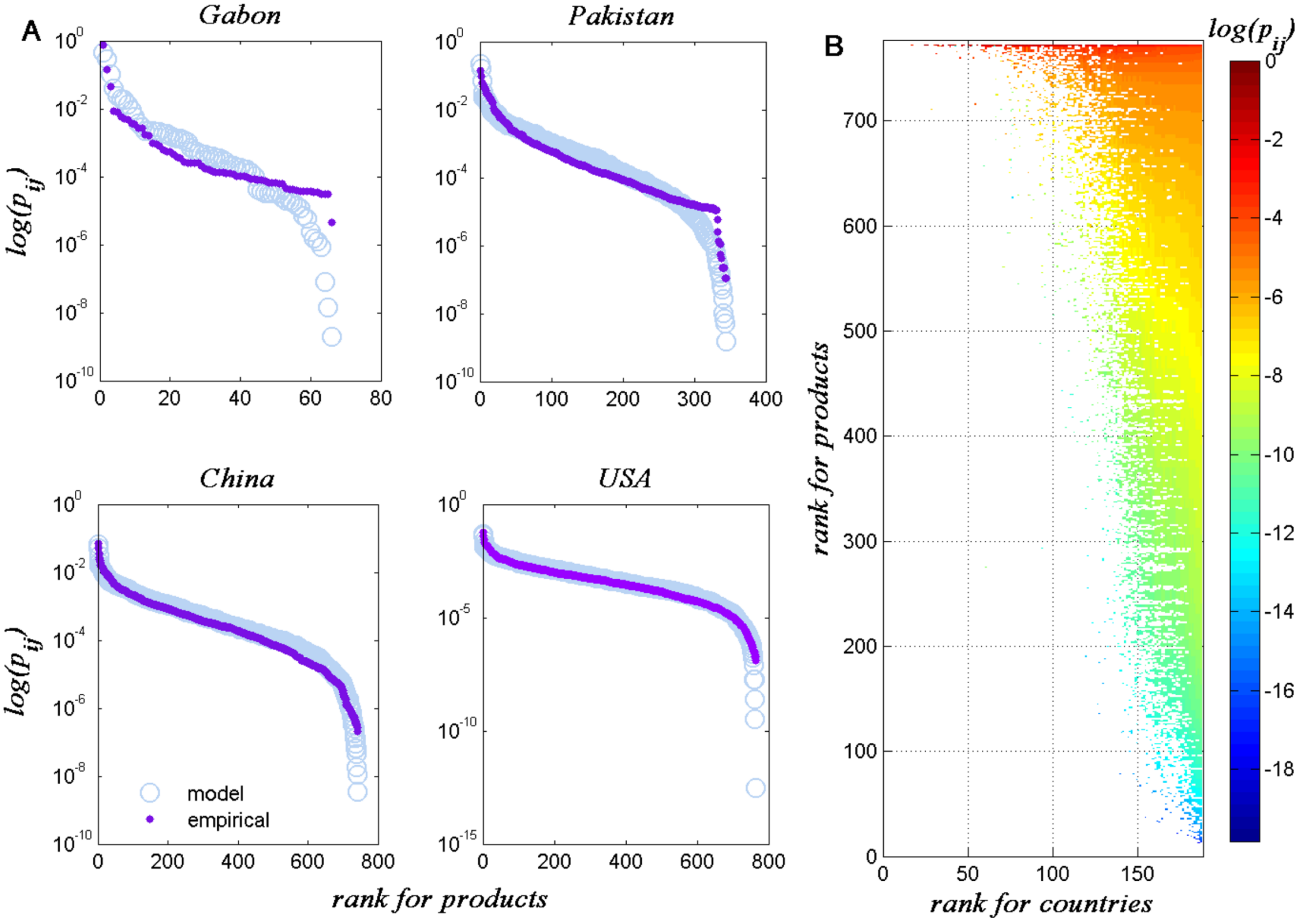
(A) Comparisons between the theoretical and the empirical *p*_*ij*_ for four selected countries. (B) The simulated “triangular structure” from Eqs (5) and (6); the colors represent ln *p*_*ij*_.

**Fig 4:**
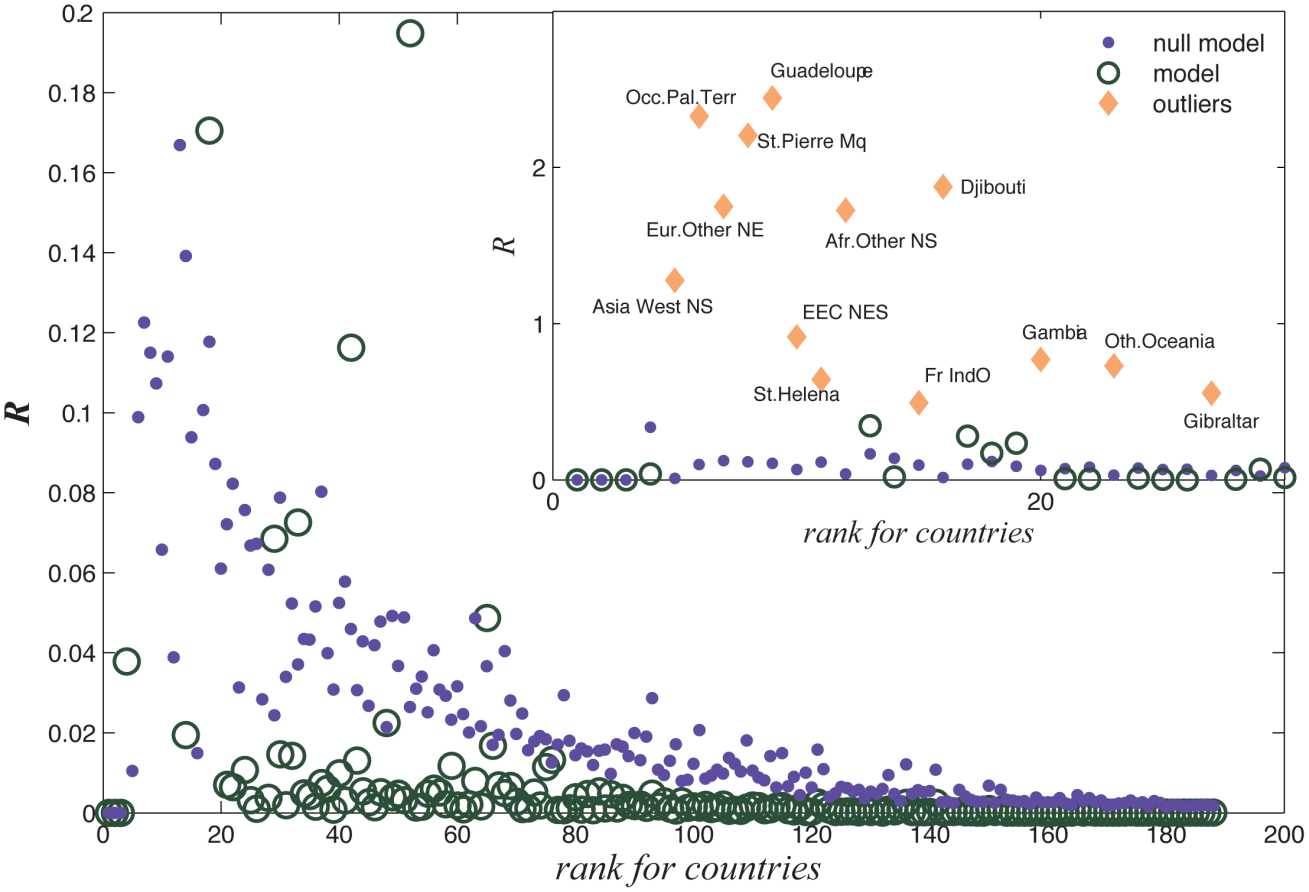
Relative entropies between the theoretical and the empirical results measured by the Kullback-Leibler Distance for all countries. Blue dots are the relative entropies for the Null model and the red hollow circles are those for the theoretical model. Insets show the outliers with very large errors (larger than 0.5).

We can solve Eqs (5) and (6) to derive the theoretical values of *p*_*ij*_ and sort them in a decreasing order to obtain the theoretical export share distribution curve. By selecting the single free parameter *k*, we can fit the empirical data for all countries (see Method section). Four countries (Gabon, Pakistan, China, and the United States) are selected to illustrate detailed comparisons. From Fig 3A, we see that the theoretical distribution curves are in good agreement with the empirical data in general. The accuracy of the theoretical analysis is much higher for large countries (China and the USA) than that of small countries. Such large deviations can be observed in the tail parts of the distributions for the small countries. To quantify the deviation precisely between the empirical distribution and the theoretical ones across different countries, we define the Kullback-Leibler (KL) distance (relative entropy) [21] for country *i* as follows

$$\begin{array}{c} R_{i}\left(p|\right|p^{*})=\frac{1}{D_{i}}\sum_{j=1}^{D_{i}}p_{ij}^{*}\;\ln\;\frac{p_{ij}^{*}}{p_{i,r(j)}}, \end{array}$$ (7)

where *p* is the theoretical distribution, p* is the empirical distribution, and *r*(*j*) is the rank order of product *j* in the theoretical curve (see Discussion section). The result is shown in Fig 4. We sort all countries by their total export value *F*_*i*_, and the horizontal axis represents the rank of a country. It is apparent that the KL distances are small for large countries. The KL distances increase as the country size decreases. For some countries such as Guadeloupe and St.Pierre Mq, our model cannot provide reasonable results (their KL distances are very large, as shown in the inset of Fig 4). The reasons for the existence of these outliers require further studies.

We compare our model with a null model, the uniform distribution on all products, *p*_*ij*_ = 1/*D*_*i*_ for any *j*. In Fig 4, the KL distance between the null model and the empirical data is indicated by the dots. We know almost all countries are below this curve, except for the outliers.

**Fig 5:**
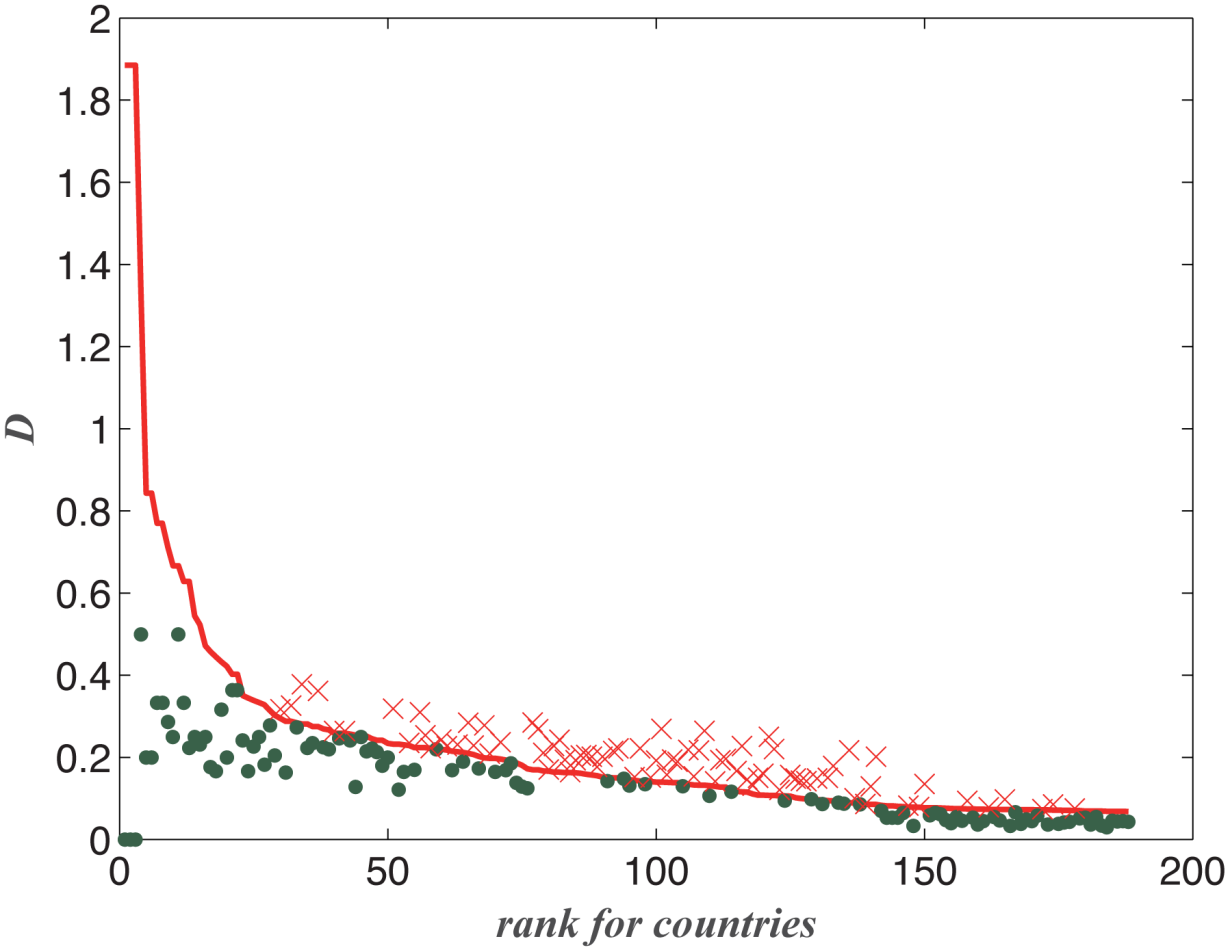
KS statistics for all countries. The red curve represents the critical KS statistics threshold of given sample size. The points above the curve marked by red crosses correspond the countries who are not passing the KS-test.

We also implement Kolmogorov-Smirnov test (KS-test) to check if *p*_*ij*_ and $p_{ij}^{*}$ are random numbers drawn from the same distribution (see Fig 5). Most countries (110 out of 180 countries) have passed KS-test in a 95% confidence level and the countries who cannot pass the test always have KS statistics very close to the critical level. The KS-test standards for these mid-size countries are always much higher than those small countries.

We attempted to use our model to reproduce the “triangular structure”, as shown in Fig 3B. The same trend that large countries export diverse products but focus on some ubiquitous products is also observed. However, the landscape of the triangle in Fig 2C is very rough (i.e. the values are not changing continuously) but it’s quite smooth and continuous in Fig 3B. The reasons behind this difference will be discussed in the following section.

## Discussion

**Fig 6:**
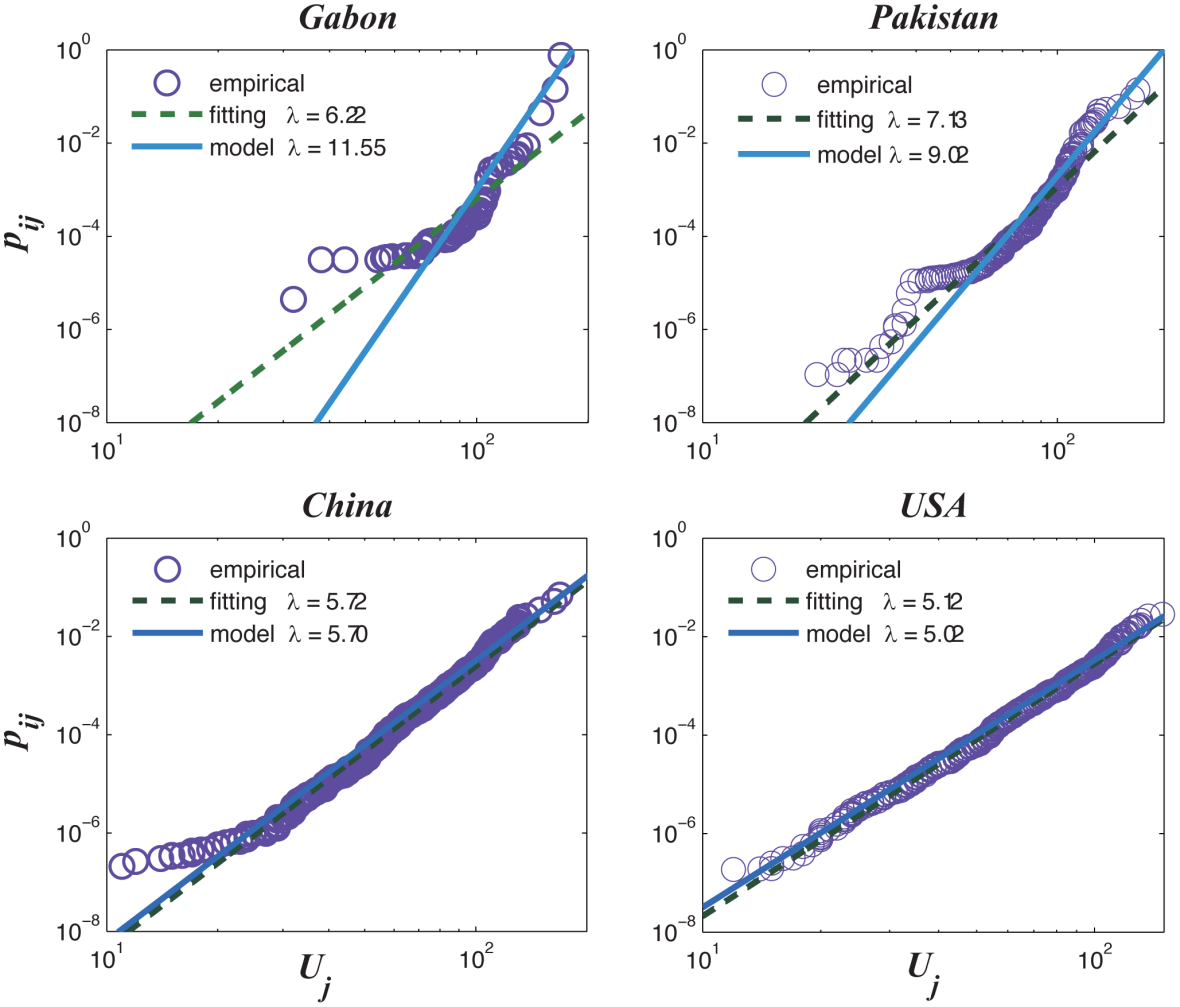
Empirical relationship between *p*_*ij*_ and *U*_*j*_ as well as the predicted exponents for four selected countries. The green dashed line represents the best fit to the empirical data.

By using the standard MEP (see method section), we can determine the model *p*_*ij*_ using Eq 5; the resulting expression is

$$\begin{array}{c} p_{ij}\propto U_{j}^{\lambda_{i}D_{i}} \end{array}$$ (8)

where λ_*i*_ is the Lagrangian multiplier for country *i* which can be computed by *D*_*i*_, *U*_*j*_. Therefore, we predict a power law dependence between *U*_*j*_ and *p*_*ij*_. Our model can fit empirical share distribution curves only if this equation holds. We tested this power law relationship by plotting the empirical $p_{ij}^{*}$ and *U*_*j*_ for four selected countries, as shown in Fig 6. The power law exponents are in good accordance with the model predictions for China, the USA, and Pakistan, except for Gabon. The small deviation for large countries of this power law relationship is the main reason why they are in much better accordance with our model than the small countries.

**Fig 7:**
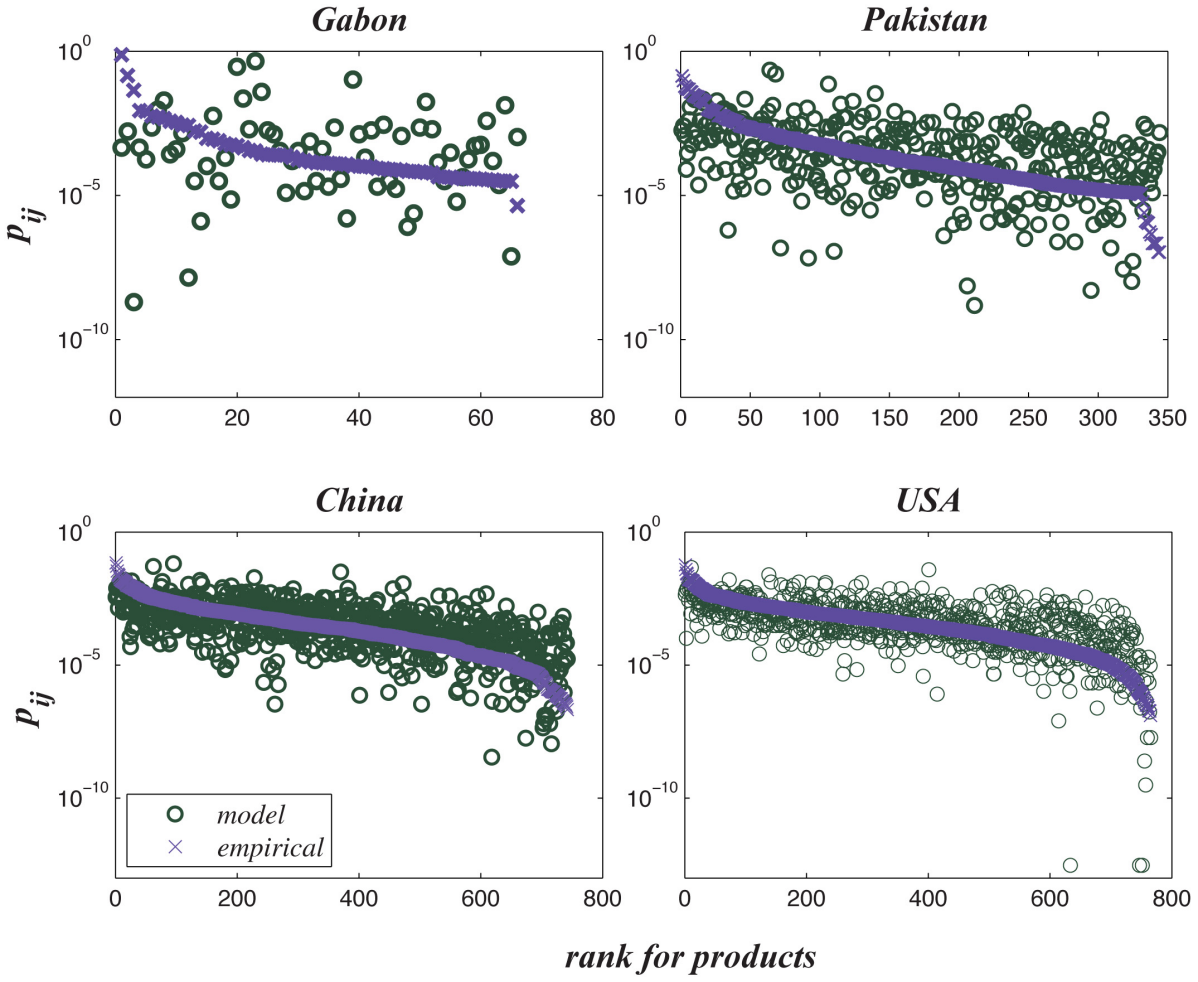
Theoretical *p*_*ij*_ with original rank orders of all products for the four selected countries.

Note that our model predicts the statistical distribution of export shares but not the specific export value for each product. That is, the rank orders of products in the predicated distribution curve are not identical to the original distribution in the empirical data (they are identical if Eq 8 holds strictly). Thus, when we plot the export shares, *p*_*ij*_, for all products in the order of the empirical rank-ordered curves in Fig 7, the points scatter around the empirical ranked curves. In addition, the deviations from the empirical curves increase as the size of countries decreases. Therefore, our model cannot predict the exact export value for each product.

**Fig 8:**
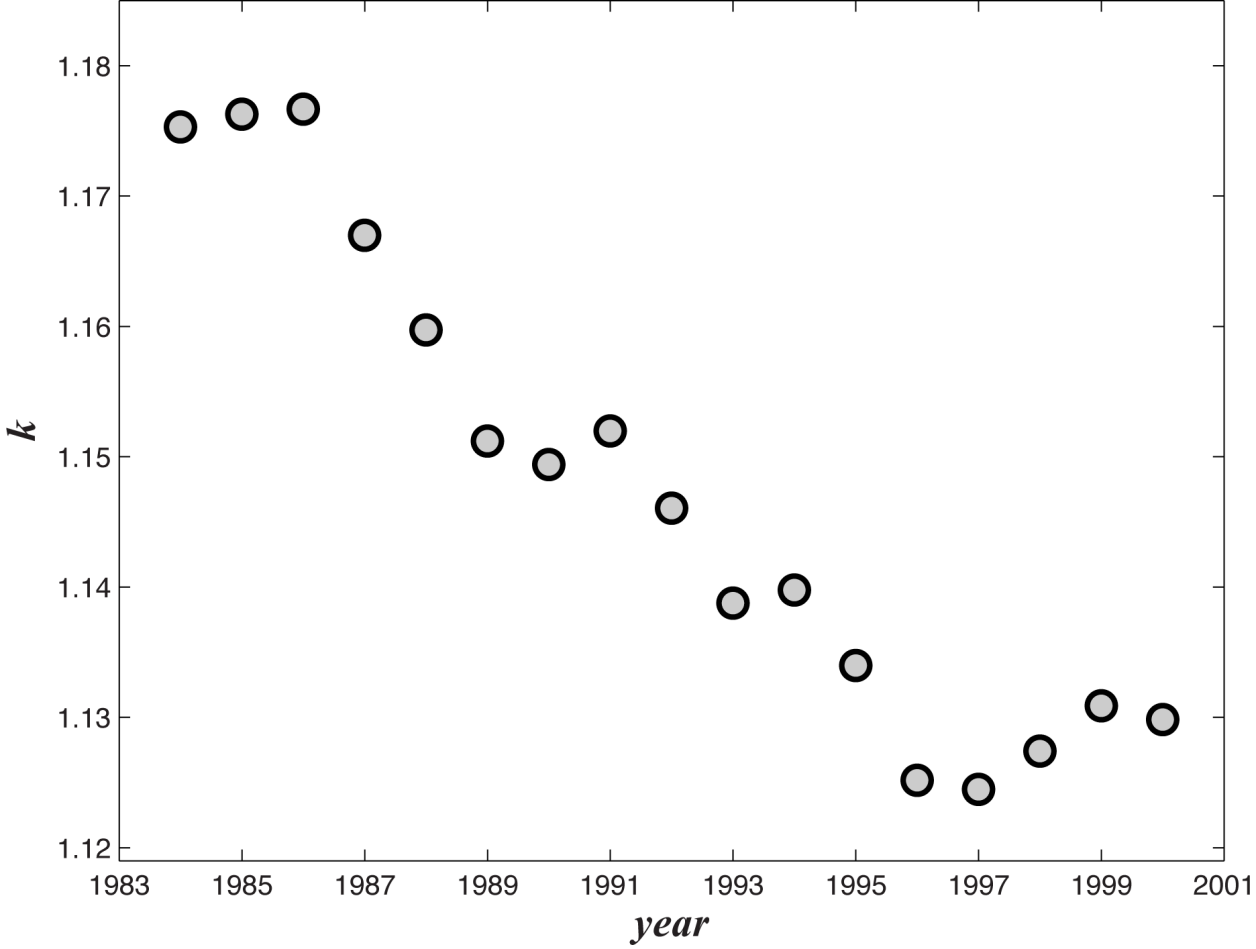
The variation evolution of *k* from 1984 to 2000.

In Eq 6, we introduced the free parameter *k*, which reflects the dependence between complexity budget and the gross level of complexity in average. Note that the value of *k* changes along time. We apply our model to all of the empirical data from 1984 to 2000 and determine the best fitted *k* plotted in Fig 8. An apparent trend that *k* almost continuously decreases is observed in the plot, which may imply the decay of the dependence of countries’ export strategies on the global market. We suppose that this trend is the reflection of the onset of globalization process approximately two decades ago in our data. As the size of the global market increases, countries are able to adjust their strategies more freely. Therefore, the complexity budget becomes less tight than before, especially for the small countries. However, this hypothesis must be tested by further research.

## Concluding Remarks

In general, this paper uncovers general patterns in international division of labor from the empirical data and attempts to attribute the patterns to simple mathematics of MEP. Although large countries have more opportunity to export various kinds of products, they may need to focus their major export shares on popular but not ubiquitous products. To explain this observation, we assume that all countries are pursuing maximum entropy on products: “putting their eggs in different baskets” as much as they can under the consideration of heterogeneity of complexity for different products measured by their logarithm of ubiquity. Determining the correct form of the constraints in the MEP framework is a difficult and subtle task. After a large number of trials, we finally find that the constraints listed in Eq 6 can work. This constraint can be explained as the complexity consumption of country *i* is assumed to balance with its complexity budget in the optimal situation. We also have tried other alternative constraints, which always lead to greater deviations.

One of the merits of our model is that only one parameter *k* is required to fit nearly all of the export share distribution curves, especially for the large countries. In particular, *k*, as a new paramount measure in international trade and division, can be regarded as the sensitivity of the division of labor strategies on the international market structure. In addition, *k* was found to decrease almost continuously during 1984 to 2000.

However, our model is limited to predicting the overall export share distribution and not the exact value of a specific product. Furthermore, all of the results are tested by only one dataset. More evidence is needed to provide more general conclusions based on our model. Nevertheless, we believe that our model of international trade can also be applied to the division of labor at other scales.

## Methods

Intuitively, the easiest approach to find the final solution of Eqs (5) and (6) is to search different values of *k* and compare *p*_*ij*_ with $p_{ij}^{*}$ because our model has only one parameter, *k*. However, this method is not likely to find the optimal strategy because all countries must be considered simultaneously in our model. In addition, it is difficult to determine a good standard to balance the closeness between the model and the empirical data for different countries simultaneously. Furthermore, the existence of outlier countries adds further complexity. Therefore, we use the following strategy to determine the best *k* to fit the curves for all countries.

**Fig 9:**
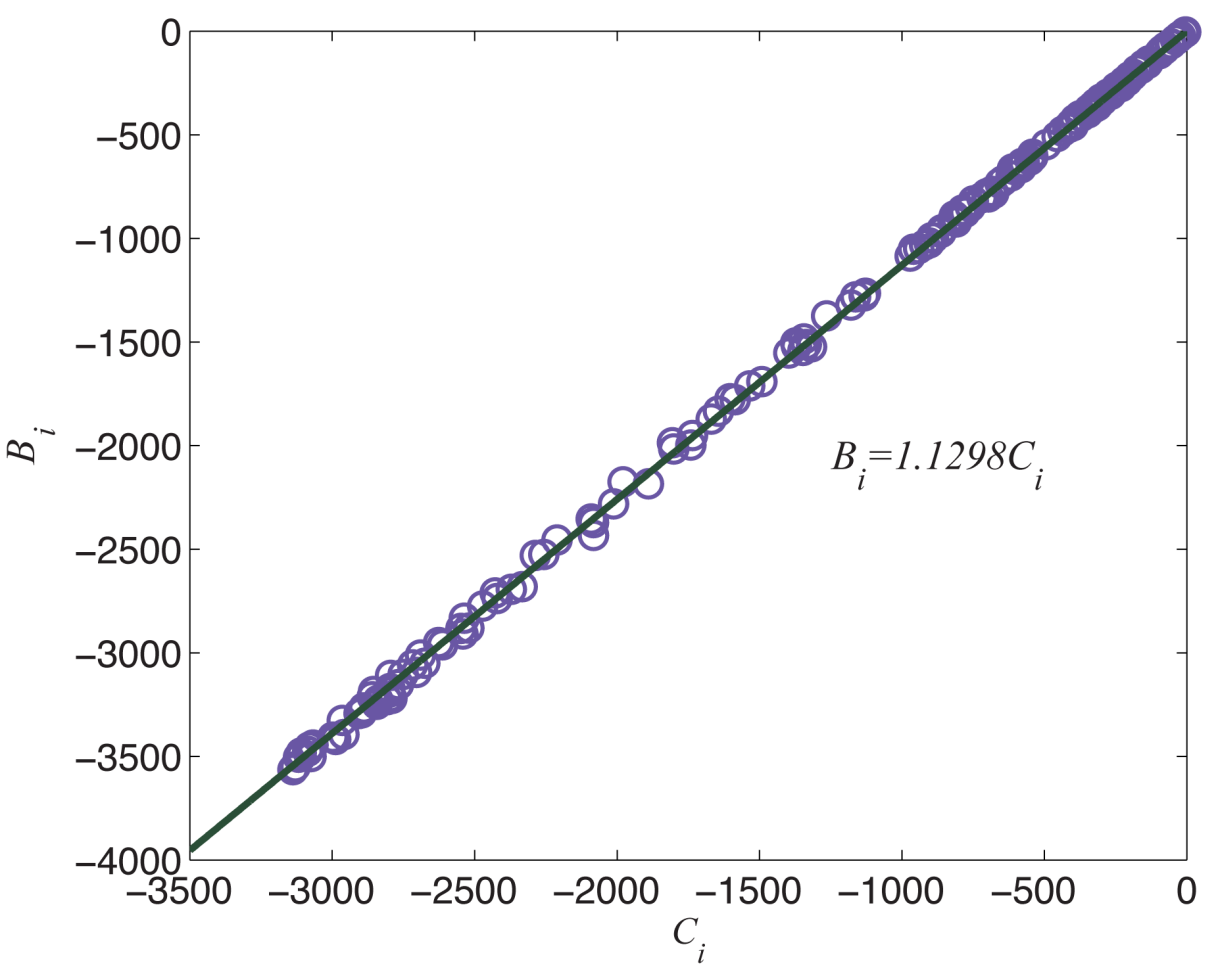
The linear relationship between *C*_*i*_ and *B*_*i*_.

We suppose that the empirical $p_{ij}^{*}$ is the optimal solution of our MEP model, that means $p_{ij}^{*}$ enables Eq 6 satisfied for all *i*. Therefore, *k* is the best estimation satisfying all equations Eq 6 for any *i* if we replace *p*_*ij*_ with $p_{ij}^{*}$. We denote $B_{i}\equiv D_{i}\sum_{j=1}^{D_{i}}p_{ij}^{*}\;\text{ln}\;\frac{1}{U_{j}}$, and $C_{i}\equiv \sum_{j=1}^{D_{i}}\;\text{ln}\;\frac{1}{U_{j}}$, and then plot all pairs of (*C*_*i*_, *B*_*i*_) on one plot. We find that data points form linear relationship as shown in Fig 9. Then we can estimate the best fit of *k* by doing linear regression for *B*_*i*_ on *C*_*i*_.

Once *k* is known, we can insert *k* into the original equation (Eq 6) to get a complete maximum entropy problem (Eqs 5, 6). It can be solved by using the standard Lagrangian multiplier method (see the 3rd section in S1 File). And the expression for *p*_*ij*_ is

$$\begin{array}{c} p_{ij}=\frac{U_{j}^{\lambda_{i}D_{i}}}{\sum_{j=1}^{D_{i}}U_{j}^{\lambda_{i}D_{i}}}, \end{array}$$ (9)

where λ_*i*_ is the Lagrangian multiplier for country *i*, which can be solved by Eq 6.

## Supporting Information

S1 File The complementary file of the manuscript. **Figure A**. Top 10 USA Exports in 2000. **Figure B**. Top Gabon Exports in 2000.
(DOCX)
