# Supplementary material for: Maximum Entropy for the International Division of Labor
Source: PLoS One. 2015 Jul 14;10(7):e0129955. doi: 10.1371/journal.pone.0129955 (PMC4718631; doi:10.1371/journal.pone.0129955)
Supplement: S1 File — Figure A. Top 10 USA Exports in 2000. Figure B. Top Gabon Exports in 2000. (DOCX) [file pone.0129955.s001.docx]

Supplementary Information for

Maximum Entropy for the International Division of Labor

Hongmei Lei, Ying Chen, Ruiqi Li, Deli He, and Jiang Zhang

School of Systems Science, Beijing Normal University, Beijing, 100875

S1. Top products for selected countries

Fig S1. Top 10 USA Exports in 2000


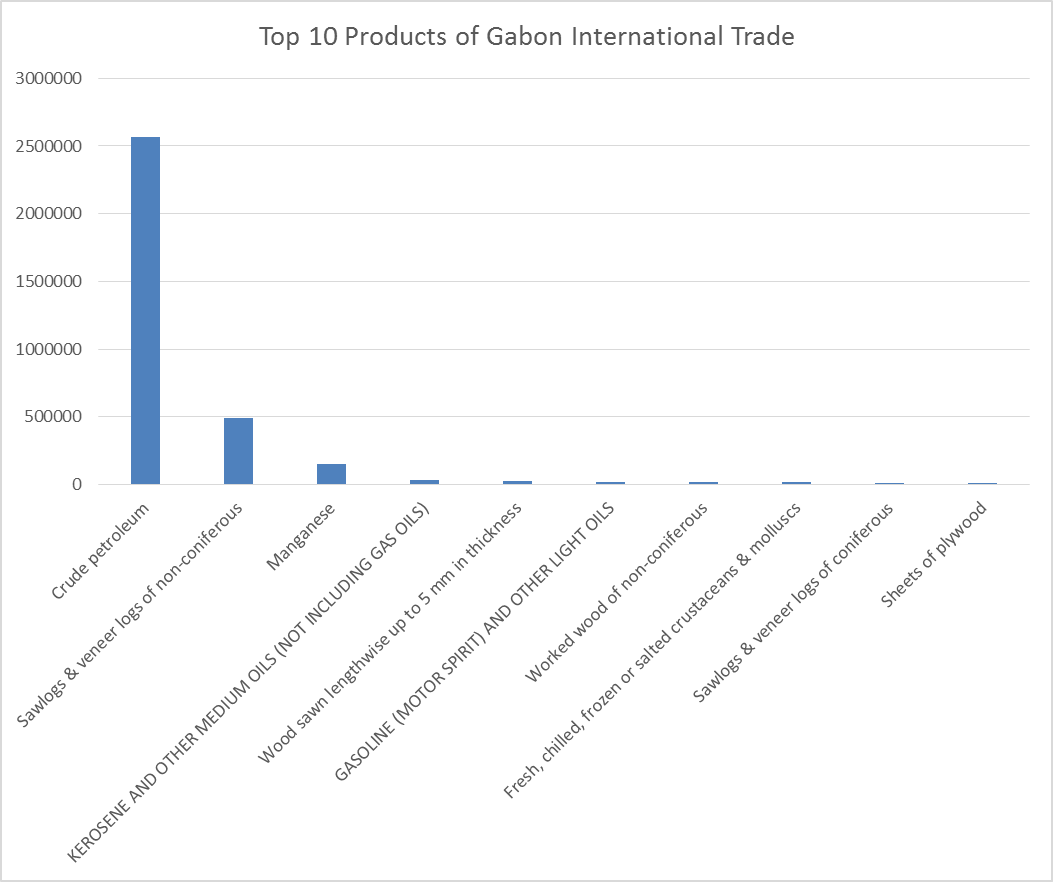


Fig S2. Top Gabon Exports in 2000

S2. Avoidance of the negative value of the complexity level

In the main text, we define the complexity level of product *j* as the negative logarithm of ubiquity *U_j_*. Because *U_j_* is greater than 1, the complexity level is less than 0. It’s a little uneasy that complexity level is a negative value. In fact, this magnitude problem doesn’t change the problem. Besides, we can avoid this problem by multiplying 1/*U_j_* a large constant (e.g. the number of countries *N*), then

$K_{j}=\ln\frac{N}{U_{j}}$ (S.1)

where *N* is the number of countries in the bipartite network. Therefore, we can guarantee *K_j_*> 0 (for any *j*), since *N > U_j_*. In addition, we will show that this modification will not alter our optimization problem (Eq. 5,6 in the main text) because the left hand of Eq. 6 in the main text is:

$D_{i}\sum_{j=1}^{D_{i}} p_{ij}\ln\frac{N}{U_{j}}=D_{i}\sum_{j=1}^{D_{i}} p_{ij}\ln N+D_{i}\sum_{j=1}^{D_{i}} p_{ij}\ln\frac{1}{U_{j}}=D_{i}\ln N+D_{i}\sum_{j=1}^{D_{i}} p_{ij}\ln\frac{1}{U_{j}}$ (S.2)

Simultaneously, the right side of Eq.6 becomes:

$k\sum_{j=1}^{D_{i}} \ln\frac{N}{U_{j}} =kD_{i}\ln N+k\sum_{j=1}^{D_{i}} \ln\frac{1}{U_{j}}$ (S.3)

Therefore, the difference between Eq. (S.2) and Eq. (S.3) is a constant:

$\text{Constant}=(k-1)D_{i}\ln N$ (S.4)

Thus, if we alter the definition of *B_i_* as

$\tilde{B}_{i}=k\sum_{j=1}^{D_{i}} K_{j} -\text{Constant}$ (S.5)

Which means the new complexity budget $\tilde{B}_{i}$ is a linear function of the gross level of complexity for all products, then the new constraint:

$D_{i}\sum_{j=1}^{D_{i}} p_{ij}\ln\frac{N}{U_{j}}=\tilde{B}_{i}$ (S.6)

implies

$D_{i}\sum_{j=1}^{D_{i}} p_{ij}\ln\frac{1}{U_{j}}=k\sum_{j=1}^{D_{i}} \ln\frac{1}{U_{j}}$ (S.7)

if we insert Eq.(S.2), Eq.(S.4), and Eq.(S.5) into Eq.(S.6). Thus, Equation (6) in the main text is recovered.
